# The Prognostic Impact of Pathology on Patients With Pseudomyxoma Peritonei Undergoing Debulking Surgery: A Systematic Review and Meta-Analysis of Retrospective Studies

**DOI:** 10.3389/fsurg.2020.554910

**Published:** 2020-11-16

**Authors:** Shengnan Zhou, Huaiyu Zhao, Xiaodong He

**Affiliations:** ^1^Department of General Surgery, Peking Union Medical College Hospital, Chinese Academy of Medical Sciences, Beijing, China; ^2^Department of Surgery, Fuwai Hospital Shenzhen Center, China Academy of Medical Science, Shenzhen, China

**Keywords:** pseudomyxoma peritonei (PMP), pathology, debulking, complete cytoreductive surgery, survival rate

## Abstract

**Background:** Pseudomyxoma peritonei (PMP) is a rare clinical condition with fatal outcomes, which is characterized by the progressive accumulation of mucinous ascites and peritoneal implants. Some studies have reported the effect of PMP biology on patient outcome. The objective of this study was to analyze published articles focusing on the impact of pathology on the prognosis of PMP patients undergoing debulking.

**Methods:** Data from all studies regarding the prognosis of patients, with different pathologies, who underwent debulking surgery were analyzed. We searched PubMed, the Wiley Online Library, Ovid, and the Cochrane Library (through January 2020). Studies were confined to those articles written in English. Five studies were identified, and the differences in 5-year survival rates were analyzed according to the Kaplan–Meier survival curves. The hazard ratios (HRs) of the 5-year survival rates were calculated.

**Results:** The mean and median 5-year survival rates of all patients were 39 and 40%, respectively. The median overall survival was 49.3 months. The mean 5-year survival rates of low-grade PMP was 45.2%. The five studies had sufficient data to calculate HRs from the 5-year survival rates data, and three had HRs lower than 1. The total HRs was 0.54, with a 95% CI between 0.33 and 0.89 (*P* = 0.01).

**Conclusions:** Among PMP patients receiving debulking surgery who are not able to undergo complete cytoreductive surgery, low-grade biological PMP had a better prognosis than high-grade PMP.

## Introduction

Pseudomyxoma peritonei (PMP) is an uncommon clinical syndrome that is featured by the accumulation and redistribution of mucinous ascites in the peritoneal cavity, usually resulting from rupture of a mucin-producing neoplasm, typically of appendiceal origin (1). The annual incidence of PMP is about 2–4 per million (2, 3). PMP is often asymptomatic in the initial stages, and as the disease burden increases, some vague abdominal symptoms may appear, such as abdominal discomfort or pain (4). The most common manifestation was acute appendicitis (27%), the second-in-line presentation was abdominal distension (23%), and the other presentations included new-onset hernia and ovarian masses in females (5). Recently, with the widespread application of cross-sectional imaging, this disease may also be diagnosed accidentally when radiographic imaging was performed (6). The disease will cause ascites, the complete obstruction of the intestinal tract, which is manifested as nausea and vomiting, and without prompt treatment, the patient will eventually die from starvation (7).

Surgery has always been the main treatment for PMP. Sugarbaker et al. (8) first introduced complete cytoreductive surgery (CRS) plus heated intraoperative intraperitoneal chemotherapy (HIPEC), which provided new ideas for PMP treatment and has been suggested as the standard therapy (9). However, a portion of patients (16.5–25.8%) (10, 11) were no longer suitable for radical surgery at the time of diagnosis due to the huge burden of PMP; for example, the massive disease invades the first porta hepatis or the caudate lobe, which is difficult to be cleaned, and the proximal third of the stomach and small bowel are involved, which would lead the length of the remnant intestines to be shorter than 2 m. For these patients, who represent a group with highly advanced PMP, the next best choice is debulking surgery (12). The definition of debulking surgery is a surgical technique that is done when the macroscopic tumor in the abdominal cavity cannot be removed. Currently, there is a clear distinction between CRS and debulking surgery according to completeness assessed by the cytoreduction score (13). CCR0 shows that there are no gross residual tumors in the abdominal cavity after surgery, CCR1 indicates that the diameter of the residual tumors is <2.5 mm, CCR2 indicates that the diameter of the residual nodules is between 2.5 mm and 2.5 cm, and CCR3 indicates that the residual nodules are larger than 2.5 cm in diameter. Generally, CCR2 and CCR3 are incomplete cytoreductions and are considered debulking surgeries with macroscopic residual tumors. Debulking surgery aims to obtain prolonged overall survival (OS) and to improve the quality of life substantially by relieving symptoms over a long period of time. Moreover, debulking surgery (14, 15) appears to be the safer approach with a relatively lower mortality and morbidity rate than CRS, which is carried out only at a fraction of centers around the world (16–18).

The pathological classification of PMP is still controversial, for the biological nature of PMP is a dynamic process of continuous change from benign to malignant disease (19). Originally, the pathology of the PMP lesions can be split into three groups (20): diffuse peritoneal adenomucinosis (DPAM), peritoneal mucinous carcinoma (PMCA), and an intermediate/discordant subtype (PMCA-I/D). When the PMP patients were treated by the same surgeon uniformly, the 5-year survival rates was 75% for the patients whose pathological type were DPAM, 50% for the PMCA-I/D subgroup, and 14% for the PMCA patients (21). In 2006, Bradley et al. (22) reviewed the outcomes of 101 patients and classified PMP into low-grade mucinous carcinoma peritonei (MCP-L), which contained DPAM and PACA-I/D, two types that did not have significantly different 5-year survival rates (68% for DPAM and 62% for PACA-I/D), and high-grade mucinous carcinoma peritonei (MCP-H). In recent studies, Nakakura et al. (1) and Chua et al. (23) also supported the Bradley criteria and considered it to be the rational classification method to predict the outcomes of PMP patients. Based on this knowledge, the Peritoneal Surface Oncology Group International (PSOGI) (9), American Joint Committee on Cancer (AJCC) (24), and World Health Organization (WHO) (25) have proposed various classification systems. To make the pathologic classification of pseudomyxoma peritonei less disputed and confusing, we can conclude that MCP-L includes DPAM and PMCA-I/D tumors, and MCP-H just includes PMCA tumors. This pathological classification method was accepted by many areas and treatment centers. However, all of these classifications, like the two-/three- and four-group classification system, were proposed based on a limited quantity of patients, and it is still far from clear which classification system could categorize PMP patients into different prognostic groups preferably.

There have been very few clinical trials, especially randomized controlled studies, documenting the relationship between biology and the prognosis of PMP patients who receive debulking surgery, as the incidence of pseudomyxoma peritonei is very low. In addition, as CRS + HIPEC has already been agreed upon as the standard therapy for PMP patients, many studies ignored that there are still some PMP patients who do not have the opportunity to receive CRS + HIPEC therapy because of the advanced disease situation or limited technology in some medical centers (26, 27). Most of these patients need to receive debulking surgery to relieve their symptoms, but whether all these patients can benefit from debulking surgery in terms of survival rates is still controversial (28), especially when these PMP patients are classified as low grade or high grade. Therefore, the significance and novelty of our work is to focus on those neglected PMP patient populations and analyze the 5-year survival rates of patients with different pathological types after receiving debulking surgery, which could be informative for physicians when making decisions.

## Methods

### Literature Search Strategy

“Pseudomyxoma Peritonei” or “Pseudomyxoma Peritonei Syndrome” and “debulking” were used as key terms to search the literatures through January 30, 2020, using the PubMed, Wiley Online Library, Ovid, and Cochrane Library databases. The included studies were limited to the published journal articles that were written in English within the past 20 years. In addition, we reviewed the references of the included literature to identify records from other sources. The entire search process was carried out independently by two blinded individuals, that is, the information that may have an impact on the literature filters was removed, such as date, the name of the journal, the author, the author's organization, and the funding status.

### Exclusion Criteria

**Figure 1:**
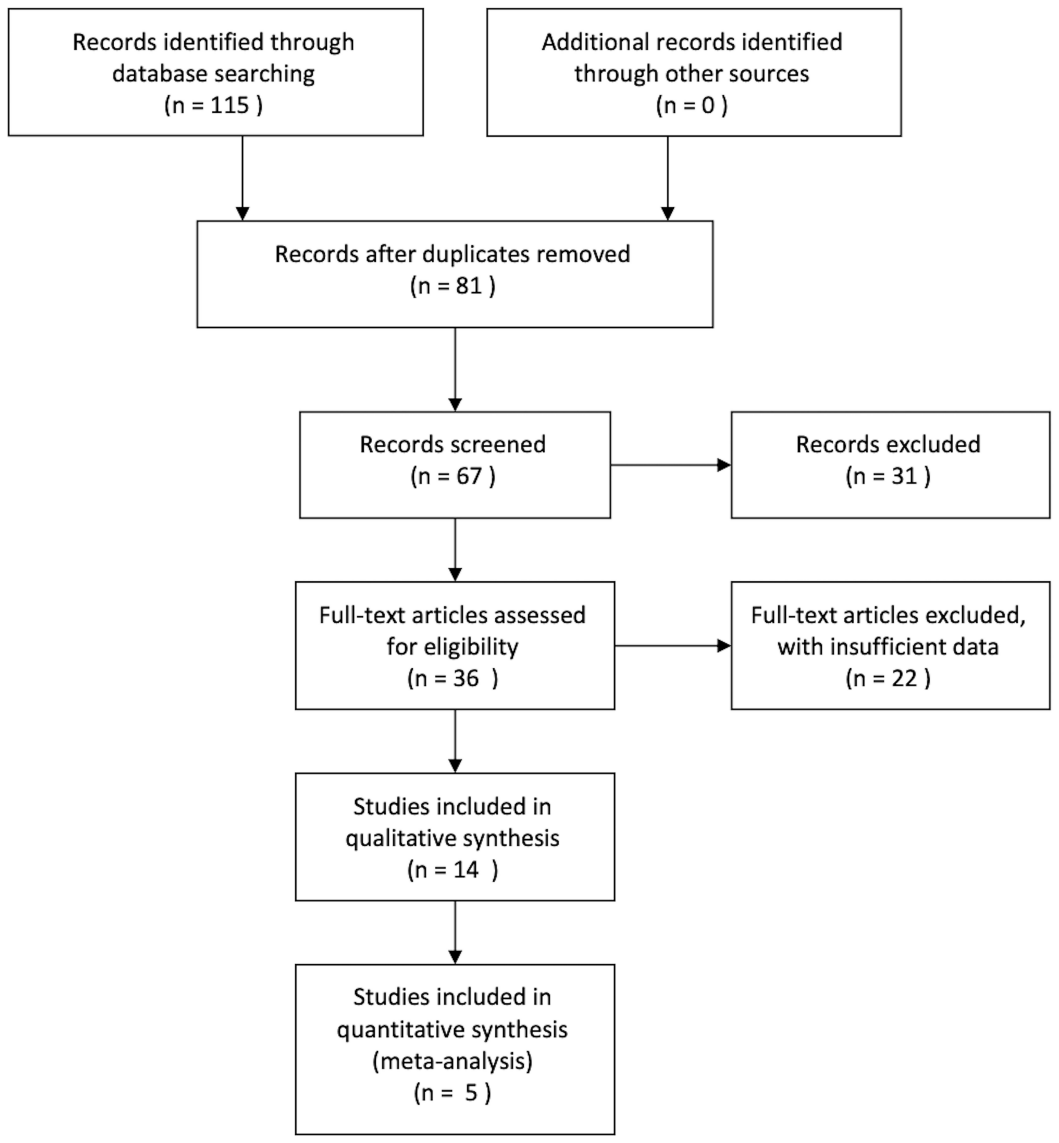
Study flow diagram.

Case reports, comments, abstracts, and review articles were excluded from our studies. Studies that had insufficient data and studies that did not stratify PMP patients who received debulking surgery based on pathology were also excluded. In addition, repeated studies were not included, such as those that included research data were released by the same research centers, and only the most recent data that were published by each center were included. In the end, eligible studies should present the relationship between biology and prognosis in patients with PMP who underwent debulking surgery. The flow chart is shown in Figure 1. Data on the 5-year survival rates and survival curves were collected.

### Data Extraction and Quality Assessment

Microsoft Excel was used to record the name of the first author, year of publication, sample size, 5-year survival rates, mortality, and median OS of all PMP patients and patients with low-grade disease. If the HRs were not given in the included study, the HRs were extracted from the survival curves with the software Engauge Digitizer by plotting points on the survival curves.

As all the included studies were retrospective studies, the quality of the included articles was assessed by the Newcastle–Ottawa scale (NOS). Three aspects were evaluated: case selection, comparability, and outcome; the scores ranged from 0 to 9 (allocated as stars). The included studies that obtained six or more stars were considered to be of high quality.

### Data Analysis

Review Manager 5.2 (Cochrane Collaboration, Oxford, UK) was used for statistical analysis. The heterogeneity was detected by the chi-square test. *P* > 0.10 was considered homogeneous; otherwise, the data were heterogeneous. In addition, *I*^2^ was applied as an indicator of heterogeneity. When *I*^2^ > 50%, the data were considered heterogeneous. In the case of homogeneity, a fixed effect model was chosen; otherwise, a random effect model was used. The extracted data were applied to calculate the HRs, and a forest plot was used to present the analysis results.

## Results

### Search Results

A preliminary search according to the determined previously searched terms identified 115 related studies. Excluding reviews, case reports, and duplicate articles, a total of 14 related studies were found. After carefully reading the original text, there were five articles that met the inclusion criteria that presented the relationship between biology and prognosis in patients with PMP who received debulking surgery. The flow diagram is shown in Figure 1. These five studies reported data on 766 patients (Table 1), and the quality of the included articles was valued by the NOS scoring system with a score between 6 and 8 points.

**Table 1:** List of information about the included studies.

**Study**	**Size (*n*)**	**5-year survival (%)**	**Median OS (m)**	**Morbidity of low-grade pseudomyxoma peritonei (PMP) (%)**	**5-year survival of patients with low-grade PMP (%)**	**Pathology method**
Andreasson et al. (29)	40	40	39.3	25	40	Bradley
Chua et al. (23)	373	24	50.4	64	33	Bradley
Dayal et al. (30)	205	30	51.9	60	38	Bradley
Delhorme et al. (14)	39	46	55.5	64	45	Ronnett
Ronnett et al. (21)	109	55		72	70	Ronnett
Total	766					
Mean		39	49.3	59	45.2	
Median		40	51.2	64	40	
**Study**	**Newcastle–Ottawa scale (NOS)**	**Type of study**	**SPIC/EPIC (%)**	**Heated intraoperative intraperitoneal chemotherapy (HIPEC) (%)**	**Systemic Chemotherapy (%)**	**Country**
Andreasson et al. (29)	7	Retrospective cohort study	77.5	17.5	23 (Preoperative)	Sweden
Chua et al. (23)	8	Retrospective cohort study	2	60	16 (Preoperative)	16 Regions
Dayal et al. (30)	7	Retrospective cohort study	0	62.9	0	UK
Delhorme et al. (14)	6	Retrospective cohort study	0	2.5	64 (post-operative) 44 (Preoperative)	France
Ronnett et al. (21)	7	Retrospective cohort study	100	0	0	US

*OS, overall survival; SPIC, sequential post-operative intraperitoneal chemotherapy*.

### The Results of the Meta-Analysis

The mean and median 5-year survival rates of all PMP patients who received debulking surgery in the five studies were 39 and 40%, respectively. The median OS was 49.3 months for these patients, and one study (21) did not report the OS of all patients. Because two included articles did not provide the date of mortality, the mean mortality rate in the remaining studies was 1.2%. Of the five studies included, the mean and median values of the proportions of low-grade PMP were 59 and 64%, respectively. The mean 5-year survival rate of the low-grade PMP subgroup was 45.2%.

The data on the 5-year survival rates or Kaplan–Meier survival curve evaluations in these five studies were sufficient to calculate HRs. The statistical heterogeneity was found among the studies (*P* = 0.0008, *I*^2^ = 79%), so a random effect model was used for the meta-analysis. Among the five studies that were investigated, the HRs of three studies were lower than 1, accompanied with confidence intervals that did not reach 1 (Chua: HR 0.66, CI 0.52–0.83; Dayal: HR 0.28, CI 0.18–0.43; Ronnett: HR 0.35, CI: 0.21–0.58).

**Figure 2:**
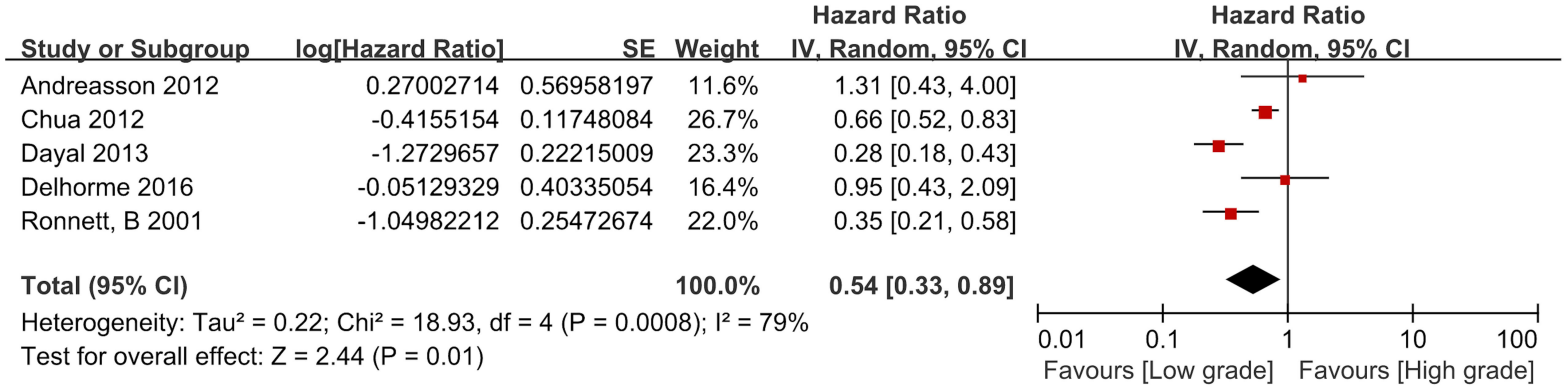
Forest plot of low-grade and high-grade pseudomyxoma peritonei.

One of the five studies had a hazard ratios (HRs) close to 1 (0.95), and its confidence intervals contained the value of 1. The remaining one study had an HR higher than 1 (1.31) with confidence intervals that included 1. The relationship between HRs and the value of 1 are shown in Figure 2. The results of the meta-analysis showed that among the PMP patients who received debulking, the prognosis of patients with low-grade pathology was better than that of patients with high-grade pathology, and there were significant differences [HR = 0.54, 95% CI (0.33, 0.89), *P* = 0.01].

### Bias Evaluation

A systematic, comprehensive, and unbiased search was conducted for all studies related to our subject to reduce sampling bias. Two individuals adopted a blinded method to select studies strictly in accordance with the inclusion and exclusion criteria to control for selection bias. A quantitative evaluation of the included literature was conducted with the NOS evaluation method to reduce research bias. Funnel plots, which are often used to evaluate publication bias, do not apply to this paper because there are only five articles, far fewer than nine articles.

## Discussion

This meta-analysis of five retrospective studies that included 766 patients and compared the 5-year survival rates of MCP-L groups and MCP-H groups who received debulking surgery showed that the 5-year survival rates of low-grade groups were significantly better. Indeed, in patients who received CRS (23), the 5-year survival rate of the low-grade group was 91% and was higher than the 68% of the high-grade group, which is in accordance with our results of the meta-analysis. This would appear to indicate that despite the experience of the centers and treatment regimens (surgical debulking or CRS), biology is also an important determinant factor of PMP patient prognosis, probably because high-grade mucinous carcinoma peritonei easily diffuses into the abdomen, resulting in an extensive burden of disease and recurrence. However, the mean 5-year survival rate of our study was 39%, which was lower than that of some centers that observed patients who derived survival and symptomatic benefits despite incomplete cytoreductive surgery (31) and found that the 5-year survival rates was close to 50% with a low morbidity rate. The proportion of high-grade disease in those patients fluctuated between 10 and 17% (32), which is far lower than the average proportion of high-grade disease (41%) of our five included studies. Therefore, we can speculate that as the proportion of high-grade pathological types among patients who underwent debulking surgery increased, the 5-year survival rates gradually decreased. This proved that PMP is definitely a heterogeneous disease, especially at the histopathology level. The pathological type determines the natural course of PMP progression and prognosis. Therefore, a “one-size-fits-all” technology for PMP patients may not be appropriate. We suggest that doctors should learn to use different treatment therapies for PMP patients with MCP-H and those with MCP-L.

In addition to pathological type and surgical treatment, there are many factors related to the prognosis of PMP patients; for example, the extent of peritoneal seeding [which can be quantified by the Peritoneal Cancer Index (PCI)] and the use of chemotherapy, such as locoregional chemotherapy, including intraperitoneal chemotherapy (IPC) and HIPEC (33), has been studied and reported in many countries and regions around the world (34–36) and can improve the 5-year survival rates, although with higher mortality and morbidity (37, 38). If there is no visible residual cancer or residual tumor deposits that measure <2.5 mm in size (CRR0 or CRR1) after surgery, chemotherapeutic drugs can penetrate into the cells and eradicate residual lesions (39, 40). However, the application of systemic chemotherapy for PMP patients is still controversial because of the peritoneal–plasma barrier (41), which affects the concentration of chemotherapy drugs in peritoneal tumor tissues. This mechanism is also the theoretical basis for intraperitoneal chemotherapy because the drug concentration in the peritoneal cavity is 20–1,000 times higher than the plasma drug concentration (42). In our five included studies, most of the patients were treated with different chemotherapy regimens. Regardless of whether systemic chemotherapy or intraperitoneal chemotherapy was used, these methods did not have much effect on 5-year survival rates. Therefore, the clinical heterogeneity caused by adjuvant chemotherapy did not interfere with the results of this meta-analysis. It can be seen from this that the size of the residual lesion is a prerequisite for ensuring the effectiveness of HIPEC. More research is still needed to explore whether HIPEC should be performed after debulking surgery to improve survival rates and quality of life, and the choice of chemotherapy drugs, the number of cycles, timing of chemotherapy, and the combined application with systemic chemotherapy also need further study.

The *I*^2^ of this study was 79%, indicating that the included studies have high heterogeneity (43), so we decided to apply a random effects model to estimate the pooled effect size and partially correct the heterogeneity. Moreover, improving the accuracy of the estimated confidence interval increases the examination of efficiency. Herein, we will discuss the sources of heterogeneity in this meta-analysis from three aspects. First, the difference in sample size may cause heterogeneity. Based on the forest plot, two outliers were found to have relatively small sample sizes (14, 29). Therefore, a sensitivity analysis was carried out by removing these two studies and indicated that the results of the meta-analysis did not change [HR = 0.41, 95% CI (0.23, 0.74), *P* = 0.03], but *I*^2^ increased to 86%; therefore, we tried to remove the study with the largest sample size (23). The results showed that HR = 0.52, 95% CI (0.27, 0.98), and *P* = 0.04, and *I*^2^ dropped to 75%. There was no difference with the original analysis results, which strengthens the credibility of the original analysis results. Second, two studies (14, 21) used the three-group pathological classification proposed by Ronnett et al., and the other three studies (23, 29, 30) used the two-group pathological classification proposed by Bradley et al. Therefore, we performed a subgroup analysis simply according to the different pathological classification methods. The results are shown in Table 2. Although there was a slight change in *I*^2^, the subgroup analysis did not reduce the heterogeneity of the meta-analysis. Therefore, it can be inferred that the pathological classification method is not the main source of heterogeneity. The last but not the least, as the subjects were PMP patients who underwent debulking surgery in different studies, the difference in the degree of debulking (CCR2 or CCR3) may be the source of heterogeneity. However, because the original texts of the included studies did not provide detailed reports on the surgical procedure, it was impossible to conduct further subgroup analyses. In summary, although the heterogeneity of this meta-analysis was high, the results were credible.

**Table 2:** Subgroup analysis according to pathology.

**Subgroup factors**	**Grouping standards**	**No. of articles**	***P***	***I*^**2**^**	**HR (95% CI)**
Pathological classification	Two-group	3	0.12	86%	0.56 (0.27, 1.16)
	Three-group	2	0.23	77%	0.55 (0.21, 1.45)
Total		5	0.01	79%	0.54 (0.33, 0.89)

Currently, with increased understanding of the disease (such as the clinical manifestation and prognosis) and improvements in surgical techniques, the mortality and complication rates of CRS + HIPEC have slowly decreased in recent years, but of course, patient selection bias cannot be ruled out (44). This has led many clinical centers to focus on selecting the optimal treatment while ignoring the insight shared by Blake Cady, MD (45) more than 20 years ago. “In the world of surgical oncology; Biology is King; Selection is Queen; Technical maneuvers are the Prince and Princess; Occasionally the prince or princess tries to usurp the throne; they almost always fail to overcome the powerful forces of the King and Queen.” Therefore, if the histopathology subtypes are not considered, just advocating bulking surgery for PMP patients could be biased. Especially for high-grade mucinous carcinoma peritonei patients, debulking surgery seems to be effective in relieving symptoms (27). For prolonging the survival rates of high-grade mucinous carcinoma peritonei patients, our meta-analysis did not support that debulking surgery is an effective treatment because the mean 5-year survival rates of these five articles is only 18.5%, far lower than the many studies that have been published (46). Although the current situation is not very optimistic, we should not abandon the discussion on the relationship between biology and treatment methods for these patients with severe illness and poor condition. There is still a lot we can do to reduce the symptoms of these patients, improve quality of life, and even extend their survival time through other care.

Despite rigorous screening and corrections, our meta-analysis has limitations. First, since there were only five articles included, there were not enough studies, and we did not draw a funnel plot to analyze publication bias. In addition, all of the included studies were retrospective studies. The reason for the little research in this area may be the insufficient sample sizes of single-center studies, and long-term survival beyond 5 years has been rarely reported in patients with PMP who are unable to receive CRS. Second, there is still a dispute regarding the pathological classification, and there is no uniform standard to classify PMP patients, even if the Bradley criteria or Ronnett criteria classification system, used by our included studies, were proposed on a limited number of patients. Thus, the best pathological classification needs to be determined by more researches to categorize patients with PMP into unique prognostic groups. Therefore, it is essential to include more studies to control bias, improve the credibility of the data, and update this meta-analysis.

## Conclusion

In conclusion, for patients with low-grade biology who cannot receive CRS + HIPEC for various reasons, debulking surgery is a suitable choice for relieving symptoms, improving quality of life, and prolonging survival. However, for high-grade PMP patients with an extensive burden of disease who are not suitable for CRS, the debulking surgery is more likely to offer a symptomatic benefit rather than improving survival rates. In addition, for the treatment of high-grade patients, we are more inclined to relieve symptoms and guarantee quality of life rather than blindly pursuing an improved survival rate.

## Data Availability Statement

All datasets generated for this study are included in the article/supplementary material.

## Author Contributions

XH and SZ participated in the design of this study. HZ and SZ carried out the study and performed the statistical analysis. SZ drafted the manuscript. All authors have read and approved the final manuscript.

## Conflict of Interest

The authors declare that the research was conducted in the absence of any commercial or financial relationships that could be construed as a potential conflict of interest.

## References

[1] Nakakura EK. Pseudomyxoma peritonei: more questions than answers. J Clin Oncol. (2012) 30:2429–30. 10.1200/JCO.2012.42.3764 22614983

[2] Smeenk RM van Velthuysen ML Verwaal VJ Zoetmulder FA. Appendiceal neoplasms and pseudomyxoma peritonei: a population based study. Eur J Surg Oncol. (2008) 34:196–201. 10.1016/j.ejso.2007.04.002 17524597

[3] Mittal R Chandramohan A Moran B. Pseudomyxoma peritonei: natural history and treatment. Int J Hyperthermia. (2017) 33:511–9. 10.1080/02656736.2017.1310938 28540829

[4] Moran BJ Cecil TD. The etiology, clinical presentation, and management of pseudomyxoma peritonei. Surg Oncol Clin N Am. (2003) 12:585–603. 10.1016/s1055-3207(03)00026-7 14567019

[5] Esquivel J Sugarbaker PH. Clinical presentation of the pseudomyxoma peritonei syndrome. Br J Surg. (2000) 87:1414–8. 10.1046/j.1365-2168.2000.01553.x 11044169

[6] Karande GY Chua WM Yiin RSZ Wong KM Hedgire S Tan TJ. Spectrum of computed tomography manifestations of appendiceal neoplasms: acute appendicitis and beyond. Singapore Med J. (2019) 60:173–82. 10.11622/smedj.2019035 31069398 PMC6482419

[7] Morera-Ocon FJ Navarro-Campoy C. History of pseudomyxoma peritonei from its origin to the first decades of the twenty-first century. World J Gastrointest Surg. (2019) 11:358–64. 10.4240/wjgs.v11.i9.358 31572561 PMC6766476

[8] Sugarbaker PH Kern K Lack E. Malignant pseudomyxoma peritonei of colonic origin. natural history and presentation of a curative approach to treatment. Dis Colon Rectum. (1987) 30:772–9. 10.1007/BF02554625 2820671

[9] Carr NJ Cecil TD Mohamed F Sobin LH Sugarbaker PH Gonzalez-Moreno S. A consensus for classification and pathologic reporting of pseudomyxoma peritonei and associated appendiceal neoplasia: the results of the Peritoneal Surface Oncology Group International (PSOGI) modified delphi process. Am J Surg Pathol. (2016) 40:14–26. 10.1097/PAS.0000000000000535 26492181

[10] Sinukumar S Mehta S As R Damodaran D Ray M Zaveri S. Analysis of clinical outcomes of pseudomyxoma peritonei from appendicular origin following cytoreductive surgery and hyperthermic Intraperitoneal Chemotherapy-A Retrospective Study from INDEPSO. Indian J Surg Oncol. (2019) 10(Suppl. 1):65–70. 10.1007/s13193-018-00870-w 30886496 PMC6397130

[11] Narasimhan V Pham T Warrier S Craig Lynch A Michael M Tie J. Outcomes from cytoreduction and hyperthermic intraperitoneal chemotherapy for appendiceal epithelial neoplasms. ANZ J Surg. (2019) 89:1035–40. 10.1111/ans.14985 30685879

[12] Chua TC Liauw W Zhao J Morris DL. Upfront compared to delayed cytoreductive surgery and perioperative intraperitoneal chemotherapy for pseudomyxoma peritonei is associated with considerably lower perioperative morbidity and recurrence rate. Ann Surg. (2011) 253:769–73. 10.1097/SLA.0b013e31820b4dba 21475018

[13] Sugarbaker PH. Cytoreductive surgery and perioperative intraperitoneal chemotherapy as a curative approach to pseudomyxoma peritonei syndrome. Tumori. (2001) 87:S3–5. 10.1053/ejso.2000.1038 11693816

[14] Delhorme JB Elias D Varatharajah S Benhaim L Dumont F Honore C. Can a benefit be expected from surgical debulking of unresectable pseudomyxoma peritonei? Ann Surg Oncol. (2016) 23:1618–24. 10.1245/s10434-015-5019-9 26678404

[15] Jarvinen P Ristimaki A Kantonen J Aronen M Huuhtanen R Jarvinen H. Comparison of serial debulking and cytoreductive surgery with hyperthermic intraperitoneal chemotherapy in pseudomyxoma peritonei of appendiceal origin. Int J Colorectal Dis. (2014) 29:999–1007. 10.1007/s00384-014-1933-8 24965858

[16] Baratti D Kusamura S Milione M Pietrantonio F Caporale M Guaglio M. Pseudomyxoma peritonei of extra-appendiceal origin: a comparative study. Ann Surg Oncol. (2016) 23:4222–30. 10.1245/s10434-016-5350-9 27352203

[17] Crawford C Janjua AZ Chandrakumaran K Moran B. Operability and early outcome in 48 Irish patients with peritoneal malignancy treated by surgery and intraperitoneal chemotherapy in a specialized centre. Surgeon. (2013) 11:30–4. 10.1016/j.surge.2012.03.002 22633149

[18] Kitai T Kawashima M Yamanaka K Ichijima K Fujii H Mashima S. Cytoreductive surgery with intraperitoneal chemotherapy to treat pseudomyxoma peritonei at nonspecialized hospitals. Surg Today. (2011) 41:1219–23. 10.1007/s00595-010-4495-6 21874418

[19] Legue LM Creemers GJ de Hingh I Lemmens V Huysentruyt CJ. Review: pathology and its clinical relevance of mucinous appendiceal neoplasms and pseudomyxoma peritonei. Clin Colorectal Cancer. (2019) 18:1–7. 10.1016/j.clcc.2018.11.007 30611664

[20] Ronnett BM Zahn CM Kurman RJ Kass ME Sugarbaker PH Shmookler BM. Disseminated peritoneal adenomucinosis and peritoneal mucinous carcinomatosis. a clinicopathologic analysis of 109 cases with emphasis on distinguishing pathologic features, site of origin, prognosis, and relationship to “pseudomyxoma peritonei”. Am J Surg Pathol. (1995) 19:1390–408. 10.1097/00000478-199512000-00006 7503361

[21] Ronnett BM Yan H Kurman RJ Shmookler BM Wu L Sugarbaker PH. Patients with pseudomyxoma peritonei associated with disseminated peritoneal adenomucinosis have a significantly more favorable prognosis than patients with peritoneal mucinous carcinomatosis. Cancer. (2001) 92:85–91. 10.1002/1097-0142(20010701)92:1<85::aid-cncr1295>3.0.co;2-r 11443613

[22] Bradley RF Stewart JHt Russell GB Levine EA Geisinger KR. Pseudomyxoma peritonei of appendiceal origin: a clinicopathologic analysis of 101 patients uniformly treated at a single institution, with literature review. Am J Surg Pathol. (2006) 30:551–9. 10.1097/01.pas.0000202039.74837.7d 16699309

[23] Chua TC Moran BJ Sugarbaker PH Levine EA Glehen O Gilly FN. Early- and long-term outcome data of patients with pseudomyxoma peritonei from appendiceal origin treated by a strategy of cytoreductive surgery and hyperthermic intraperitoneal chemotherapy. J Clin Oncol. (2012) 30:2449–56. 10.1200/JCO.2011.39.7166 22614976

[24] Valasek MA Pai RK. An update on the diagnosis, grading, and staging of appendiceal mucinous neoplasms. Adv Anat Pathol. (2018) 25:38–60. 10.1097/PAP.0000000000000178 29016471

[25] Reu S Neumann J Kirchner T. [Mucinous neoplasms of the vermiform appendix, pseudomyxoma peritonei, and the new WHO classification]. Pathologe. (2012) 33:24–30. 10.1007/s00292-011-1542-z 22179200

[26] Youssef H Newman C Chandrakumaran K Mohamed F Cecil TD Moran BJ. Operative findings, early complications, and long-term survival in 456 patients with pseudomyxoma peritonei syndrome of appendiceal origin. Dis Colon Rectum. (2011) 54:293–9. 10.1007/DCR.0b013e318202f026 21304299

[27] Funder JA Jepsen KV Stribolt K Iversen LH. Palliative surgery for pseudomyxoma peritonei. Scand J Surg. (2016) 105:84–9. 10.1177/1457496915598759 26232048

[28] Jarvinen P Jarvinen HJ Lepisto A. Survival of patients with pseudomyxoma peritonei treated by serial debulking. Colorectal Dis. (2010) 12:868–72. 10.1111/j.1463-1318.2009.01947.x 19519686

[29] Andreasson H Graf W Nygren P Glimelius B Mahteme H. Outcome differences between debulking surgery and cytoreductive surgery in patients with pseudomyxoma peritonei. Eur J Surg Oncol. (2012) 38:962–8. 10.1016/j.ejso.2012.07.009 22809859

[30] Dayal S Taflampas P Riss S Chandrakumaran K Cecil TD Mohamed F. Complete cytoreduction for pseudomyxoma peritonei is optimal but maximal tumor debulking may be beneficial in patients in whom complete tumor removal cannot be achieved. Dis Colon Rectum. (2013) 56:1366–72. 10.1097/DCR.0b013e3182a62b0d 24201390

[31] Chua TC Baker B Yan TD Zhao J Morris DL. Palliative effects of an incomplete cytoreduction combined with perioperative intraperitoneal chemotherapy. Am J Clin Oncol. (2010) 33:568–71. 10.1097/COC.0b013e3181b9cf47 20019578

[32] Iversen LH Rasmussen PC Hagemann-Madsen R Laurberg S. Cytoreductive surgery and hyperthermic intraperitoneal chemotherapy for peritoneal carcinomatosis: the Danish experience. Colorectal Dis. (2013) 15:e365–72. 10.1111/codi.12185 23458368

[33] Esquivel J. Technology of hyperthermic intraperitoneal chemotherapy in the United States, Europe, China, Japan, and Korea. Cancer J. (2009) 15:249–54. 10.1097/PPO.0b013e3181a58e74 19556912

[34] Elias D Gilly F Quenet F Bereder JM Sideris L Mansvelt B. Pseudomyxoma peritonei: a French multicentric study of 301 patients treated with cytoreductive surgery and intraperitoneal chemotherapy. Eur J Surg Oncol. (2010) 36:456–62. 10.1016/j.ejso.2010.01.006 20227231

[35] Oemrawsingh A de Boer NL Brandt-Kerkhof ARM Verhoef C Burger JWA Madsen EVE. Short-term complications in elderly patients undergoing CRS and HIPEC: a single center's initial experience. Eur J Surg Oncol. (2019) 45:383–8. 10.1016/j.ejso.2018.10.545 30409441

[36] Somashekhar SP Prasanna G Jaka R Rauthan A Murthy HS Karanth S. Hyperthermic intraperitoneal chemotherapy for peritoneal surface malignancies: a single institution Indian experience. Natl Med J India. (2016) 29:262–6. 28098079

[37] Kojimahara T Nakahara K Shoji T Sugiyama T Takano T Yaegashi N. Identifying prognostic factors in Japanese women with pseudomyxoma peritonei: a retrospective clinico-pathological study of the Tohoku gynecologic cancer unit. Tohoku J Exp Med. (2011) 223:91–6. 10.1620/tjem.223.91 21263209

[38] Goslin B Sevak S Siripong A Onesti J Wright GP Melnik M. Outcomes of cytoreduction with hyperthermic intraperitoneal chemotherapy: our experience at a Midwest community hospital. Am J Surg. (2012) 203:383–6. 10.1016/j.amjsurg.2011.09.009 22226143

[39] Beckert S Struller F Grischke EM Glatzle J Zieker D Konigsrainer A. [Surgical management of peritoneal surface malignancy with respect to tumour type, tumour stage and individual tumour biology]. Zentralbl Chir. (2016) 141:415–20. 10.1055/s-0033-1350857 24241953

[40] Jacquet P Averbach A Stuart OA Chang D Sugarbaker PH. Hyperthermic intraperitoneal doxorubicin: pharmacokinetics, metabolism, and tissue distribution in a rat model. Cancer Chemother Pharmacol. (1998) 41:147–54. 10.1007/s002800050721 9443628

[41] Jacquet P Sugarbaker PH. Peritoneal-plasma barrier. Cancer Treat Res. (1996) 82:53–63. 10.1007/978-1-4613-1247-5_4 8849943

[42] Lu Z Wang J Wientjes MG Au JL. Intraperitoneal therapy for peritoneal cancer. Future Oncol. (2010) 6:1625–41. 10.2217/fon.10.100 21062160 PMC3076138

[43] Higgins JP Thompson SG Deeks JJ Altman DG. Measuring inconsistency in meta-analyses. BMJ. (2003) 327:557–60. 10.1136/bmj.327.7414.557 12958120 PMC192859

[44] Rodt AP Svarrer RO Iversen LH. Clinical course for patients with peritoneal carcinomatosis excluded from cytoreductive surgery and hyperthermic intraperitoneal chemotherapy. World J Surg Oncol. (2013) 11:232. 10.1186/1477-7819-11-232 24040889 PMC3850876

[45] Cady B. Basic principles in surgical oncology. Arch Surg. (1997) 132:338–46. 10.1001/archsurg.1997.01430280012001 9108752

[46] Glehen O Mohamed F Sugarbaker PH. Incomplete cytoreduction in 174 patients with peritoneal carcinomatosis from appendiceal malignancy. Ann Surg. (2004) 240:278–85. 10.1097/01.sla.0000133183.15705.71 15273552 PMC1356404

